# Herbicide resistance in grass weeds: Epigenetic regulation matters too

**DOI:** 10.3389/fpls.2022.1040958

**Published:** 2022-11-10

**Authors:** Madhab Kumar Sen, Katerina Hamouzová, Pavlína Košnarová, Amit Roy, Josef Soukup

**Affiliations:** ^1^ Department of Agroecology and Crop Production, Faculty of Agrobiology, Food and Natural Resources, Czech University of Life Sciences Prague, Prague, Czechia; ^2^ Excellent Team for Mitigation (E.T.M.), Faculty of Forestry and Wood Sciences, Czech University of Life Sciences Prague, Praha, Czechia

**Keywords:** abiotic stresses, DNA methylation, epigenetic regulations, herbicide resistance, histone modification, phenotypic plasticity

## Abstract

Although herbicides have been successfully used for controlling weeds, their continuous use has developed in the evolution of resistance to all major herbicide modes of action worldwide. Reports suggest that the members of Poaceae family are more prone to developing herbicide resistance than other families. In plants, epigenetic mechanisms play critical roles by increasing their stress-adaptive potential in a rapidly changing environment. Epigenetic mechanisms involve alteration of the expression of genetic elements, but without any changes in the DNA sequence. Although the possible roles of epigenetic mechanisms in contributing to survival and fitness under various stresses are well documented in model plants and crops, their contribution to herbicide resistance in weeds is still in its infancy. A few studies with herbicides have shown differential expression of DNA methyltransferases, histone methyltransferases and DNA demethylases in response to the herbicides; however, no further studies were conducted. In the case of herbicide stress, exploring how these epigenetic processes affect the gene expression pattern in individual plants subjected to recurrent selection would be exciting. Hence, our mini-review will focus on the potential contributions of epigenetic mechanisms to the adaptive responses of grass-weedy species to herbicide stress. A better understanding of these epigenetic changes will add novel perceptions to our knowledge of herbicide resistance evolution in weeds enabling the development of herbicides with novel targets.

## Introduction

Regardless of motionlessness, plants have developed phenotypic plasticity to deal with various biotic and abiotic stresses. The information content of the genome and its synchronized expression in response to stress are crucial for the adaptability of the plants, especially when they are subjected to various unavoidable environmental stresses. In addition to various transcription factors (triggered by signal transduction), epigenetics is also one of the major factors contributing to the variability in stress response (Sahu et al., 2013; Munshi et al., 2015; Ashapkin et al., 2020). Epigenetic mechanisms (such as covalent modifications of the DNA and histones) include all the amendments of gene expression that ensue without altering the DNA sequence. In a broad sense, epigenetically-modified mechanisms can be classified into three main types: chromatin rearrangement and histone post-translational modifications (PTMs), DNA methylation and transcriptional and post-transcriptional level modifications of stress-responsive genes triggered by non-coding RNAs (ncRNAs). A detailed description of the individual mechanisms can be found in Mozgova et al., 2019 and Chinnusamy and Zhu, 2009. The epigenetic modifications might generate naturally, genetically or environmentally within an organism. Generally, the stress-induced epigenetic adjustments occur in a randomized manner and are expected to get restored soon after their occurrence. However, under certain circumstances, they might endure more, thereby providing a ‘stress memory’(Bhar et al., 2022). Such ‘stress memories’ are sometimes inheritable (Markus et al., 2018). Besides this, plants are likely to flower and produce seeds before normal when exposed to stresses as a part of the species conservation strategy (Yaish et al., 2011). Hence, in these cases, the seeds might transmit the accrued epigenetic information of the stressed plants to their progenies, eventually leading to adaptive evolution (a phenomenon known as epigenetic transgenerational memory (Molinier et al., 2006). Evidences of probable contributions of various epigenetic mechanisms to stress responses and memory in plants are rising and will continue to increase in future (Kinoshita and Seki, 2014). Thus, it might be exciting to contemplate that several perceived transgenerational responses to stress conditions could be ascribed to various epigenetic mechanisms.

Currently, herbicide resistance is a significant challenge in modern agriculture, with new cases reported yearly (Powles and Yu, 2010; Chauhan, 2020). Even though herbicide resistance cases have been reported on numerous plant families, reports suggest that grasses are more prone to developing herbicide resistance than other families. Grasses have their predominance as weeds in general (88 species as of 7^th^ June 2022; https://www.weedscience.org). The most troublesome weed species of the Poaceae family include *Alopecurus myosuroides*, *Apera spica-venti*, *Avena fatua, Echinochloa* spp., *Eleusine indica*, *Lolium* spp. etc. Epigenetic regulatory mechanisms such as DNA methylation, histone modification, and non-coding RNA (ncRNA) activities are expected to contribute to the phenotypic plasticity and adaptative capacity in various plant species, particularly in the weedy grasses. Even though the possible roles of epigenetic mechanisms in contributing to survival and fitness under various stresses are well documented in model plants and crops, their contribution to herbicide resistance in weeds is yet to be fully explored.

The development of herbicide resistance in grass weeds is an excellent illustration of the adaptableness of plant species to abiotic stress, making it an appealing topic for many evolutionary biologists. Exploring the epigenetic modifications and interpretation of the results is a critical challenge, especially in the case of herbicide resistance, where most species are non-model. An amalgamation of epigenetic regulations with the transcriptional changes (resulting in herbicide resistance) might divulge the exact mechanism by which epigenetic regulations orchestrate the gene expression in response to the herbicide treatment. Therefore, the current mini-review will emphasize the basis of epigenetic regulation in plants and will provide an updated overview of the probable consequences of epigenetic changes on herbicide resistance. This review will also briefly discuss the techniques that can be used to study the epigenetic modifications involved in herbicide resistance.

## Epigenetic regulation in adaptation of grass weedy species to the herbicide stress

The most important resistance mechanisms to herbicides include target-site resistance (TSR) and non-target-site resistance (NTSR). TSR includes DNA mutations leading to structural changes to herbicide-binding sites and/or overexpression of target proteins, whereas NTSR includes enhanced detoxification and/or decreased herbicide absorption and translocation (Gaines et al., 2020; Sen et al., 2021). In contrast to the herbicide TSRs, NTSR mechanisms involve complex biochemical and molecular processes and are far less understood (Délye, 2013; Ghanizadeh and Harrington, 2017). Currently, NTSR *via* enhanced detoxification is considered of particular importance because of their potential to confer unpredictable resistance to multiple mechanisms of action, including herbicidal compounds that are yet to be discovered. Several herbicides cause oxidative stress, similar to plant abiotic stresses. Enhanced herbicide detoxification involves detoxifying enzymes such as cytochrome P450 monooxygenases (P450s), glutathione transferases (GSTs), ATP-dependent [ATP-binding cassette (ABC)] transporters and a few other enzymatic systems involved in detoxifying xenobiotics (Délye et al., 2015; Gaines et al., 2020). Hence, most of the enzymes involved in herbicide metabolization are also linked with the basal stress response pathways in plants (Radwan, 2012; Markus et al., 2018).

**Figure 1:**
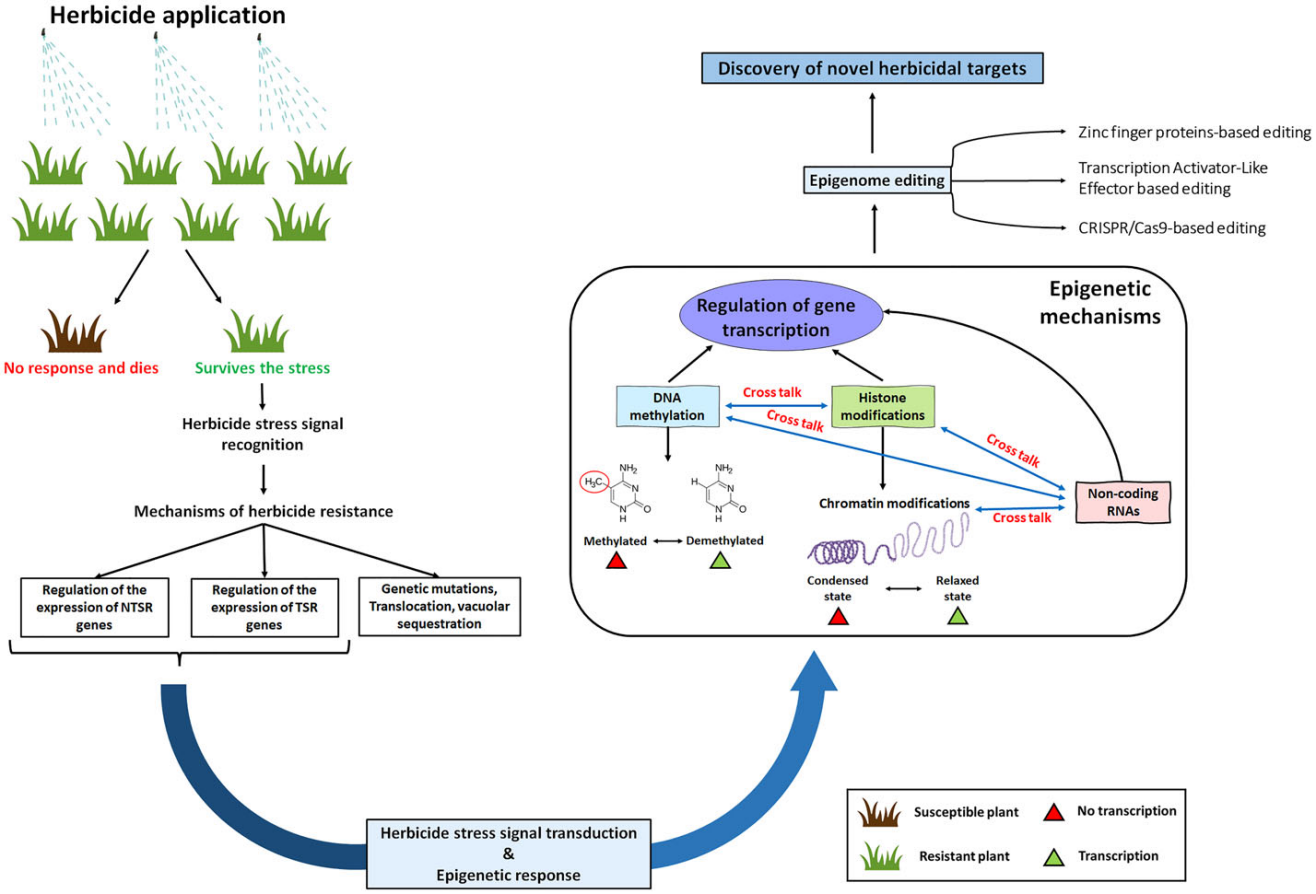
A schematic diagram showing the scope of epigenetic mechanisms in herbicide resistance. Plants can survive the herbicide stresses either by target site mechanisms (TSRs) or by non-target site mechanisms (NTSRs). Following the herbicide stress signal recognition and signal transduction, there might be an epigenetic response which might cause modifications in the DNA and histone methylation enzymes. Additionally, these modifications might also occur in the epigenetic regulators such as small RNAs and histone variants. Finally, these epigenetic mechanisms will regulate the expression of TSR genes (such as *acetolactate synthase* or *acetyl CoA Carboxylase*) and NTSR genes (such as *cytochrome P450s*, *Glutathione S-transferases* and *ABC transporters*).

Epigenetic mechanisms are known to modify gene expression patterns due to various biotic and abiotic stresses (Arıkan et al., 2018; Ashapkin et al., 2020). As a result of epigenetic effects, it can be assumed that a gene might get provisionally flipped on. This might result in overproduction of the herbicide binding site (such as *ALS*, ACCase or EPSPS) or genes that encode metabolism or sequestration (such as cytochrome P450s, *GST*s or *ABC transporter*s) (**Figure 1**). On the contrary, epigenetic effects might also result in provisional flipping off a gene that converts a pro-herbicide to its active form. In a study conducted by Pan et al., 2022, they discovered that differential methylation of CpG islands of the *CYP81A68* promoter occurred between resistant (R) and susceptible (S) plants. They concluded that epigenetic regulation might play a role in acetolactate synthase (ALS) and Acetyl-CoA carboxylase (ACCase)-inhibiting the resistance evolution of *E. crus-galli* (Pan et al., 2022). In a study with *Conyza canadensis*, the researchers found differential methylation patterns between the R and S plants (Margaritopoulou et al., 2018). Similarly, another study with atrazine in rice indicated differential regulation of methyltransferases in response to the herbicide (Lu et al., 2016). When treated with different glyphosate concentrations, a significant increase in global DNA methylation has been detected in wheat (Nardemir et al., 2015). After that, several studies with herbicides have shown differential expression of DNA methyltransferases, histone methyltransferases and DNA demethylases in response to the herbicides. However, no further follow-up studies were conducted. Besides studying only the differential methylation patterns, the focus must also be turned toward their localization. The gene expression dynamics are expected to vary with the localization of methylation in the genome. Previously, DNA methylation was often described to have only ‘silencing’ effects. However, with advancements in the genome-scale mapping of methylation, it was discovered that the function of DNA methylation seems to vary with genomic contexts such as transcriptional start sites, within the gene body, at the regulatory regions etc. For example, genes with methylated promoters tend to show lower expression levels; hence, they are predominantly specialized for tissue-specific expression patterns (Zhang et al., 2018; Akhter et al., 2021).

Besides DNA methylation, histone modifications (such as methylation and acetylation) also play essential roles in regulating eukaryotic gene expression. Chromatin organization inside the nucleus have long been proposed to be linked with epigenetic-modifying enzymes. In plants, chromatic organization play an essential role during differentiation and stress adaptation (Baba et al., 2019; Sun et al., 2020). Synchronization in the chromatin structure and nuclear organization changes is crucial to ascertain critical heritable gene expression patterns in response to stress signals. Histone modifications, chromatin remodeling complexes, and histone variants (H2A, H2B, H3, H4 and H1) contribute to organizing DNA within the nucleus. Various studies have shown that PTMs on different histone residues might show a variety of effects on gene transcription. However, compared to acetylation, histone methylation and demethylation have relatively slow turnover. Hence methylation and demethylation of histones play more significant roles in contributing to past stress memory (Mozgova et al., 2019; Li et al., 2022). Several evidences suggest involvement of several histone methyltransferases, demethylases, acetyltransferases and deacetylases in plant stress responses. For example, members of the RPD3/HDA1 family were found to regulate gene expression through histone deacetylation in *Arabidopsis* (Yuan et al., 2013). Likewise, HDA6 was involved in drought stress tolerance and jasmonic acid signalling pathways (Murfett et al., 2001; Aufsatz et al., 2007; Yuan et al., 2013). Even though in plants, investigations with histone modifications have been done in response to a wide range of abiotic stresses, their effects in response to herbicide stress are yet to be studied. Histone acetyltransferases use acetyl-CoA as a substrate to acetylate histone lysine residues (Berndsen and Denu, 2008). ACCase is one of the most critical herbicidal modes of action, and cases of resistance against these herbicides are increasing globally. Hence, studies aiming to discover the possible roles of acetylation and deacetylation might open up new areas of research, which might result in discovering new herbicidal targets. **Table 1** shows the list of epigenetics-based research works related to herbicide sensitivities.

**Table 1:** The list of epigenetics-based research works related to herbicide sensitivities.

Species	Herbicide	Comments	Reference
*Triticum aestivum*	Glyphosate	Different glyphosate concentrations elevated the levels of global DNA methylation	Nardemir et al., 2015
Rice	Atrazine	DNA methyltransferases, histone methyltransferases and DNA demethylases	Lu et al., 2016
*Arabidopsis thaliana*	Glyphosate	Following the herbicide injury, the authors found 9,205 differentially methylated regions across the genome.	Kim et al., 2017
*Conyza canadensis*	Glyphosate	Sodium bisulfite sequencing detected differential methylation patterns between the S and R plants.	Margaritopoulou et al., 2018
*Arabidopsis thaliana*	Glyphosate, imazethapyr and 2,4-D	The results showed that following the herbicide treatment, the herbicides could change specific epigenetic pathways and regulate the expression of specific genes.	Markus et al., 2021
*Echinochloa crus-galli*	Penoxsulam, cyhalofop-butyl and metamifop	Analysis of the *CYP81A68* gene promoter identified an efficient region with differential methylation of CpG islands between S and R plants.	Pan et al., 2022

Beside playing independent roles in chromatin-mediated transcription, in some complex scenarios, histone modifications act conjunctionally in a context-dependent manner (a phenomenon known as crosstalk between histone post-translational modification). Since the first well-characterized example of histone crosstalk (stimulation of acetyltransferase activity by prior phosphorylation; Cheung et al., 2000; Lo et al., 2000), this phenomenon is currently known to be highly conserved among eukaryotes. With more evidence in recent years, it has become apparent that epigenetic modifications do not function alone but together in various combinations (Molina-Serrano et al., 2013). These combinations are hypothesized to diversify their functional states (especially during response to various stresses). These crosstalks among modifications might be categorized into two major categories: the first, histone modifications that take part in a sequential cross-talk. In sequential cross-talk, one modification either promotes or inhibits the accumulation of another modification. The second category comprises modifications that take part in a combinatorial manner. In this modification, multiple modifications work simultaneously to regulate a particular molecular response (Lee et al., 2010; Molina-Serrano et al., 2013). Since the evolution of herbicide resistance resembles an abiotic stress response, there might be possibilities for such complex epigenetic regulatory networks. Further investigations in this research area might lead to the discovery of novel herbicidal modes of action. Apart from the epigenetic modifications, the focus must also be turned toward the possibilities of involvement in various cell signaling pathways and their crosstalk with epigenetics. Signal transduction pathways are an integral part of many stress-adaptation responses. There are also some evidences where the signaling pathways might directly impact critical components of the epigenetic machinery (De Santa et al., 2007; Arzate-Mejía et al., 2011; Baba et al., 2011). Although many model plants have already described a substantial variety of signal transduction pathways and their response to various stress, nothing is known about the crosstalk between numerous signal transduction and their consequential amendments in herbicide application. There might be diversities of epigenetic mechanisms (not discovered yet) involved in response to herbicide stress which might have unexpected connections with different signaling pathways to establish transcriptional responses. Exploring the relationship between cell signaling and epigenetics might open up a large area of research that might help us comprehend the complex process by which a cell can induce transcriptional changes in response to herbicide stress signals.

## Techniques used to decrypt the epigenetic modifications involved in herbicide resistance

Recent understanding of the epigenetic function of plant chromatin is highly influenced by chromatin immunoprecipitation assay (ChIP assay) and ChIP-Seq. ChIP is an antibody-based technology used to explore the protein-DNA interaction within the cell. ChIP-Seq merges ChIP assay with next-generation sequencing technologies and is very useful for examining genome-wide-specific histone modifications. These methods can identify histone modifications along with the target of the histone modifiers (Park, 2009; Pellegrini and Ferrari, 2012). In the context of herbicide resistance, ChIP assay and ChIP-Seq can be used to understand the mechanisms of expression of the herbicide-resistance target and their related genes. Besides ChIP assays, Hi-C technique (with well-developed assembly pipelines) must also be considered by weed molecular biologists as a method of choice to characterize three-dimensional genome folding. Alongside Hi-C, 3C-Seq and Capture-C might also be useful to analyze various chromatin interactions. However, while studying *in-situ* chromatin interactions, Hi-C technique is well-preferred over the others (Grob, 2022). In general, Hi-C combines 3C, and next-generation sequencing (NGS) approaches to discover the genome-wide chromatin interactions within the nucleus (Kong and Zhang, 2019). This technology might be considered as the beginning of 3D genomics. Another well-advanced technology HiChIP (combining HiC with ChIP) is also yet to be tested to elucidate the possible roles of epigenetic processes in herbicide resistance.

Alongside histone modifications, DNA methylation is another fundamental epigenetic mechanism. Traditional molecular techniques such as PCR or cloning methods cannot be applied to detect DNA methylations. This is mainly because methyl groups are not copied during PCR amplification. Hence, this method requires a pretreatment process on the intact methylated DNA strand (Sulewska et al., 2007; Halabian et al., 2021). The most common techniques used to identify these DNA methylations are restriction enzyme digestion-based techniques, bisulfite method, shotgun bisulfite-sequencing, Methylated-DNA immunoprecipitation (MeDIP) etc. The basic principle of this type of method is that there are specific endonucleases, such as HpaII and SmaI, which can differentiate between the unmethylated and the methylated sequences. For example, HpaII digest only the unmethylated CpG in the CCGG sequences, while another enzyme MspI, cannot differentiate between the methylated and the unmethylated sequences. Hence it digests both the sequences. Some useful restriction enzyme digestion-based techniques include Methylation-sensitive amplified fragment length polymorphism (MS-AFLP), Differential methylation hybridization (DMH), Comprehensive high-throughput arrays for relative methylation (CHARM), HpaII tiny fragment enrichment by ligation-mediated PCR (HELP) etc. (Halabian et al., 2021).

The bisulfite method is based on the principle that under denaturing conditions, sodium bisulfite (an antichlor) converts only cytosines, but not 5’-methyl cytosines, into uracil. Thereafter, the uracil converts into thymine upon PCR amplifying the bisulfite-treated DNA. Finally, the bisulfite-treated DNA is analyzed by PCR and sequencing. Shotgun bisulfite-sequencing is a modified approach to the bisulfite method. In this method, the bisulfite treatment of the genomic DNA is followed by next-generation sequencing technology. The converted sequences are subsequently mapped to the reference genome sequence to spot the methyl-cytosines (Krueger et al., 2012; Halabian et al., 2021). Bisulfite sequencing is capable of delivering robust results specifically with low amounts of moderate quality template DNA and also allows higher CpG coverage with single-base resolution as compared to the other methods (Tanić, 2020). Moreover, bisulfite sequencing can also be used for analysis of repetitive sequences, which are difficult to study with microarrays due to their high chances of cross-hybridizing (Cokus et al., 2008). Despite of its preciseness and reproducibility, bisulphite-based methods are highly dependent on the bisulphite conversion of all unmethylated cytosine into uracil. Inadequate conversions might complicate the post-sequencing data analysis, particularly in plants as they have complicate genomes which are expected to have a high level of 5mC (Grunau et al., 2001; Wang et al., 2011).

MeDIP is an immunocapturing approach that uses 5-methylcytosine-specific monoclonal antibodies. This technique is used for unbiased detection of methylated DNA. The precipitated DNA is then investigated by PCR or whole genome tiling microarrays (Jacinto et al., 2008; Mohn et al., 2009). A modified version of MeDIP is MeDIP-seq. The MeDIP is coupled with next-generation sequencing technologies such as 454 pyrosequencing or Illumina in this approach. However, the accuracy of the results obtained from the MeDIP-seq must be validated with quantitative PCR (Halabian et al., 2021). In addition to these techniques, in recent years, plant molecular biologists have widely performed nanopore sequencing (Schatz, 2017; Ni et al., 2021). However, their applications in weed herbicide resistance research are yet to be tested. Nanopore sequencing has enabled researchers to identify several DNA and RNA base modifications at single nucleotide resolution, besides providing several benefits over traditional bisulfite sequencing for methylation analysis (such as more genomic coverage even with lower GC bias, higher number of CpG positions at lower read depts, considerably faster data analysis along with higher experimental reproducibility). Even though bisulfite sequencing is the most extensively used method for methylation profiling, due to short-read sequencing, methylation profiling in the repetitive genome regions remains undervalued in this technique. Additionally, there is a high chance of loss of sequencing diversity with bisulfite sequencing, which might be resolved by nanopore sequencing.

## Current challenges in weed epigenetics and the way forward

Epigenetic studies in model species are primarily based on high-resolution genomic analyses in controlled experiments. On the contrary, in the case of non-model organisms, the unavailability or non-availability of high-resolution genomic data impedes identifying the connection of epigenetic variants with phenotypic effects. The main concern for weed epigenetics in the upcoming years is to generate higher-resolution genomic data and tools in more realistic scenarios (narrowing down the gap between the laboratory-based data and field experiments). In this context, we discussed some crucial techniques that might be used to decipher the epigenetic modifications in weedy species. Additionally, high-resolution genomic analyses on a broader diversity of systems must also be considered. Genome sequencing initiatives need to be taken to facilitate weed epigenetic studies. This is essential since the effects of epigenetics are likely to fluctuate with varying genomic features and ploidy levels among species. Comparison of the nature of resistance across species with differing genomic features and ploidy levels might enable the researchers to identify the key factors driving the rate and nature of adaptation (Kreiner et al., 2018). While it is still debatable that polyploidy plays an essential role in facilitating adaptation to herbicide stress, an experiment with hexaploidy and diploid wild oat (*Avena fatua*) suggested that the effect size of a TSR mutation was much smaller in the hexaploid than in a diploid *A. fatua* (Yu et al., 2013). Even though the finding suggests that better masking of beneficial mutations may limit polyploid adaptation, such hypotheses demand more investigations (case by case). Thus, herbicide resistance, specifically through NTSR, might entail general or herbicide-specific epigenetic mechanisms. Following the application of sublethal herbicide doses, the triggered primary and/or secondary signals might provoke changes in specific pathways, which involve genes essential for herbicide resistance (*P450*s, *GST*s and *ABC transporters*). Although much remains to be explicated regarding epigenetics and herbicide resistance, there is a high possibility that the species-herbicide-specific changes leading to resistance development might be partially arbitrated by epigenetic control over gene expression.

Moreover, it is challenging to determine if epigenetics is the cause or a consequence of herbicide adaptation. Transcriptional studies with treated and untreated resistant and susceptible plants are expected to provide hints to such questions. However, it is important to validate such a hypothesis. The current situation demands the discovery of a novel herbicidal mode of action. Hence, we recommend combining transcriptomic and epitranscriptomic sequencing results (multi-omics approach). To date, no such initiatives have been taken to elucidate the mechanisms of herbicide resistance. Hence, we urge weed molecular biologists to come together and produce such multi-omics data. We think this will help us come up with some alternative solutions to the increasing problem of herbicide resistance.

Another interesting area of research might be to develop clustered regularly interspaced short palindromic repeats (CRISPR) and its associated endonuclease (Cas) epigenetic editors (Jogam et al., 2022). Besides CRISPR, Zinc finger proteins, Transcription Activator-Like Effectors (TALEs) might also be employed for genome and epigenome editing. But, CRISPR/Cas9 genome editing is predominantly preferred over the others due to its efficiency in design for any genomic targets and easy prediction concerning the off-target sites (Gupta et al., 2019). Moreover, CRISPR/Cas9 genome editing allows multiplexing and hence the researchers can target multiple genes at a time using a single construct. Epigenome editing has shown some promising results for enhancing plants’ tolerance to various environmental stresses as well as in gene functional studies (De Melo et al., 2020; Shin et al., 2022). However, yet this area of research is completely unexplored to discover the possible mechanisms of how weeds are able to escape from the herbicide stress. Till date, very few cases of targeted histone manipulation and targeted DNA methylation are reported in model plants such as *Arabidopsis* (with very specific loci and most of the loci remain to be investigated). Hence, despite of the fact that epigenome editing is well-thought-out technology for control of gene expression, supplementary researches are needed for the accurate manipulation of epigenetic marks. Additionally, most of the epigenetic marks might be unstable in plants. Therefore, efforts must be given to identify stable and heritable epigenetic mechanisms over generations. Nevertheless, despite of its detailed knowledge gap (even with the model plants), “editing” the epigenomic features might enable the researchers to elucidate the exact biological role of an epigenetic modification in herbicide stress.

## Conclusions and future directions

Rapid evolution of herbicide resistance *via* varieties of herbicide resistance mechanisms (primarily *via* metabolic resistance) is a real challenge for current weed management strategies and herbicide use. Moreover, the availability of herbicides with limited modes of action further complicates the scenario. Though the preexisting genetic variations in populations might explain a part of the plants’ adaptation strategy to abiotic stresses, several studies have shown that epigenetic modifications-mediated generation might explain most of the remaining half. Abiotic stress-induced epigenetic changes are expected to provide an adaptive advantage to an organism and generate more stable phenotypes (such as herbicide-resistant weeds). Furthermore, evidence that herbicides can trigger epigenetic responses in model plants such as rice and *Arabidopsis* shows that even sublethal herbicide doses might act similarly to other abiotic stresses. Additionally, owing to their highly dynamic nature, stable epigenetic effects following the herbicide treatment will enhance the development of herbicide resistance in weeds, specifically NTSR. This evolution of adaptive responses of grass-weedy species to herbicide stress is most likely species-specific. Thus, an updated and detailed understanding of the contribution of epigenetic modifications in phenotypic plasticity is essential to understanding how weed populations are developing resistance against the existing herbicides. Nonetheless, this is an exciting area of study that undoubtedly demands further investigations and the engagement of more weed scientists and geneticists. Moreover, owing to their dynamic nature, epigenomes respond to more environmental influences than genomes (or the stable DNA sequence). Identifying the molecular mechanisms and the signaling pathways responsible for this dynamic nature is a promising area of research, which might give valuable answers to the relationship between the epigenome and cell signaling. Challenges such as limited genome availability must be resolved to facilitate molecular and epigenetic work on weeds.

## Author contributions

MS and AR conceived the idea. MS, KH, and PK drafted the manuscript. AR and JS edited the manuscript. MS revised the manuscript. All authors contributed to the article and approved the submitted version.

## Funding

This work was supported by grants from the National Agency for Agricultural Research project (QK1820081). The salary for AR is obtained from grant “EXTEMIT - K,” No. CZ.02.1.01/0.0/0.0/15_003/0000433 and EVA 4.0,” No. CZ.02.1.01/0.0/0.0/16 019/0000803 supported by OP RDE.

## Acknowledgments

The authors thank Mr. Rohit Bharati (Department of Crop Sciences and Agroforestry, The Faculty of Tropical AgriSciences, Czech University of Life Sciences Prague) for consultations and technical assistance.

## Conflict of interest

The authors declare that the research was conducted in the absence of any commercial or financial relationships that could be construed as a potential conflict of interest.

## Publisher’s note

All claims expressed in this article are solely those of the authors and do not necessarily represent those of their affiliated organizations, or those of the publisher, the editors and the reviewers. Any product that may be evaluated in this article, or claim that may be made by its manufacturer, is not guaranteed or endorsed by the publisher.
